# Poisoning by Antipyrin

**Published:** 1891-02-21

**Authors:** 


### TOXICOLOGY.

### POISONING BY ANTIPYRIN.

We reported not long ago a nearly fatal case of poisoning by an overdose of antifebrin in the person of a Russian lady. It is true that the dose was such that the result was no more than might have been expected, but the fact remains that the use of antifebrin and phenacetin calls for caution, and that of all this class of drugs, antipyrin is by far the safest. Yet the existence of individual susceptibility or idiosyncracies must not be lost sight of even in ordering antipyrin, as is shown by a case reported in *Centralb. f. d. ges. Therapie.*, in which a lady, aged 22, having taken 15 grains of antipyrin after dinner, was seized in the course of a few minutes with pain in the back of the head, dizziness, and ringing in the ears; soon followed by palpitation, dyspnœa, cold perspiration, and a peculiar subjective sensation of warmth over the whole right side of the body and of cold on the left side. After about twenty minutes there was disturbance of vision and amaurosis lasting over half-an-hour. Hyperæmia of the optic nerve and injection of the conjunctivæ were observed. Her lips were blue, her pulse 200 in the minute, her respiration rapid and laboured, and speech impaired. In an hour and a-half an erythematous eruption appeared on the right side of the body—that which had felt warmer, and later still, severe vomiting came on. The treatment consisted in $1\frac{1}{2}$ grain doses of caffeine every three hours, and, *aut post aut propter hoc*, she improved gradually during the evening, being next day in her usual health. In the Russian case of antifebrin poisoning the effects of the drug were far graver; indeed, we are convinced that the treatment employed her recovery was wholly due, *i.e.*, to the warmth, friction, subcutaneous injections of ether, and finally and especially, the slow subcutaneous injection by gravitation only of large quantities of the physiological solution of salt in warm water; a procedure which, though rarely practised, deserves earnest consideration as one of the most powerful stimulants of a failing heart, and, as Sahli has shown, an equally energetic aid to the elimination of poisons whether ingested or produced within the organism in disease. Since the action of antipyrin as a nerve sedative in migraine, &c. is often manifested after a smaller dose, and it is always possible that a patient may be one of the susceptible ones, it would be well to begin with five grain doses, repeated once or twice if necessary; certainly 15 grains should not be given at once to a patient on the first occasion.

